# Critically ill patients with COVID-19-associated acute kidney injury treated with kidney replacement therapy: Comparison between the first and second pandemic waves in São Paulo, Brazil

**DOI:** 10.1371/journal.pone.0293846

**Published:** 2023-11-03

**Authors:** Farid Samaan, Rafaela Andrade Penalva Freitas, Renata Viana, Lívia Gâmbaro, Karlla Cunha, Tales Dantas Vieira, Valkercyo Feitosa, Eric Aragão Correa, Alexandre Toledo Maciel, Sylvia Aranha, Eduardo Atsushi Osawa, Roberta Pillar, Elias Marcos da Silva Flato, Renata Cristina da Silva, Elisa Carneiro, Fabrizzio Batista Guimarães de Lima Souza, Paula Regina Gan Rossi, Munira Bittencourt Abud, Henrique Pinheiro Konigsfeld, Riberto Garcia da Silva, Ricardo Barbosa Cintra de Souza, Saurus Mayer Coutinho, Miguel Ângelo Goes, Bárbara Antunes Bruno da Silva, Dirce Maria Trevisan Zanetta, Emmanuel Almeida Burdmann

**Affiliations:** 1 Grupo Hapvida-NotreDame Intermédica, São Paulo, SP, Brazil; 2 Instituto Dante Pazzanese de Cardiologia, São Paulo, SP, Brazil; 3 Secretaria de Estado da Saúde de São Paulo, São Paulo, SP, Brazil; 4 Imed Research Group, Hospital São Camilo Pompéia, São Paulo, SP, Brazil; 5 Unidade Assistencial Hospital Ipiranga, São Paulo, SP, Brazil; 6 Hospital São Francisco, Cotia, SP, Brazil; 7 Hospital Leforte Liberdade, São Paulo, SP, Brazil; 8 Hospital Municipal Vereador Jose Storopolli, São Paulo, SP, Brazil; 9 Hospital SEPACO, São Paulo, SP, Brazil; 10 Universidade Federal de São Paulo, São Paulo, SP, Brazil; 11 Faculdade de Saúde Pública, Universidade de São Paulo, São Paulo, SP, Brazil; 12 Laboratório de Investigação Médica (LIM 12), Faculdade de Medicina, Universidade de São Paulo, São Paulo, SP, Brazil; Universitas Pelita Harapan, INDONESIA

## Abstract

**Introduction:**

This study aimed to compare the characteristics and outcomes of critically ill patients with COVID-19-associated acute kidney injury (AKI) who were treated with kidney replacement therapy (KRT) in the first and second waves of the pandemic in the megalopolis of Sao Paulo, Brazil.

**Methods:**

A multicenter retrospective study was conducted in 10 intensive care units (ICUs). Patients aged ≥18 years, and treated with KRT due to COVID-19-associated AKI were included. We compared demographic, laboratory and clinical data, KRT parameters and patient outcomes in the first and second COVID-19 waves.

**Results:**

We assessed 656 patients (327 in the first wave and 329 in the second one). Second-wave patients were admitted later (7.1±5.0 vs. 5.6±3.9 days after the onset of symptoms, p<0.001), were younger (61.4±13.7 vs. 63.8±13.6 years, p = 0.023), had a lower frequency of diabetes (37.1% vs. 47.1%, p = 0.009) and obesity (29.5% vs. 40.0%, p = 0.007), had a greater need for vasopressors (93.3% vs. 84.6%, p<0.001) and mechanical ventilation (95.7% vs. 87.8%, p<0.001), and had higher lethality (84.8% vs. 72.7%, p<0.001) than first-wave patients. KRT quality markers were independently associated with a reduction in the OR for death in both pandemic waves.

**Conclusions:**

In the Sao Paulo megalopolis, the lethality of critically ill patients with COVID-19-associated AKI treated with KRT was higher in the second wave of the pandemic, despite these patients being younger and having fewer comorbidities. Potential factors related to this poor outcome were difficulties in health care access, lack of intra-hospital resources, delay vaccination and virus variants.

## Introduction

Coronavirus disease 2019 (COVID-19) was declared a pandemic by the World Health Organization (WHO) in March 2020 [1]. There have been 765,222,932 confirmed cases and 6,921,614 deaths worldwide as of May 3, 2023. At the same time, there have been 37,449,418 confirmed cases with 701,494 deaths reported to the WHO in Brazil [2].

Acute kidney injury (AKI) stands out as a COVID-19 complication due to its high incidence, impact on outcomes and economic burden. COVID-19-associated AKI incidence ranges from 4 to 36%, depending on study methodology [3]. Among critically ill COVID-19 patients, AKI occurs in up to 53% of cases, with approximately 26% requiring KRT [3]. AKI is an independent risk factor for death among COVID-19 patients, and the risk increases in parallel with AKI severity [4, 5]. In the first pandemic wave, the lethality of KRT-dependent COVID-19-associated AKI (KRT-AKI) reported in the majority of studies was 51 to 79% but was higher than 90% in resource-limited and vulnerable Brazilian areas [6–12].

This scenario is even more worrying when we consider the challenges and burden faced by the health care system against the pandemic over time [13]. AKI in hospitalized patients is associated with an almost two times greater length of hospitalization and costs, therefore consuming a large amount of human, infrastructural and economic resources [14, 15]. Whereas low- and middle-income countries suffered from a shortage of basic health care resources and the collapse of their health care systems in the second wave of the pandemic, high-income countries reported a reduction in in-hospital COVID-19 patient lethality over the same period of time [16, 17].

Considering the lack of studies comparing KRT-treated COVID-19-associated AKI patients in the first and second pandemic waves, especially in low- and middle-income countries, the aim of this study was to compare the characteristics and outcomes of COVID-19-associated AKI patients treated by KRT in the first and second pandemic waves in the Sao Paulo megalopolis, Brazil.

## Methods

### Design, study location and population

This was a retrospective cohort, observational, multicenter study conducted in the intensive care units (ICU) of 10 public or private hospitals in the metropolitan region of São Paulo city. The selection of participating hospitals was based on convenience sampling and included public (teaching and non-teaching) and private hospitals (owned and not owned by health insurance companies).

The inclusion criteria were ICU admission, age ≥ 18 years, diagnosis of SARS-CoV-2 infection, and COVID-19-associated AKI treated with KRT. The exclusion criteria were KRT-dependent chronic kidney disease before hospitalization and exclusive palliative care. We compared the periods of April-August 2020 and March-June 2021 because they were the peaks of the first and second COVID-19 waves in Brazil, respectively [2]. Results from the first wave of the pandemic were previously published and included 13 hospitals [9]. As three of these centers did not send patients information from the second wave of the pandemic for the present study, we included only the ten hospitals with data available from the first and second waves.

The study followed the principles of the Declaration of Helsinki and it was approved by the research ethics committees of the participating centers under certificate number 31693820.8.1001.5485. The ethics committees waived the requirement for informed consent once the study was retrospective and the guarantee of fully anonymized data was provided by the authors. All data was received in anonymized form between 02/18/2022 and 01/06/2023.

### Variables assessed

Demographic data, comorbidities, COVID-19 symptoms, date of symptom onset, vital signs, and laboratory tests at hospital admission were obtained.

Hypertension was defined by the presence of a diagnosis in the medical record or by the use of antihypertensive drugs. Diabetes mellitus was defined by the presence of a diagnosis in the medical record or by the use of oral antidiabetic drugs or insulin. Chronic kidney disease, heart failure, chronic liver disease, and chronic obstructive pulmonary disease were defined by the presence of a diagnosis in the medical record. Coronary artery disease was defined by a positive history of acute myocardial infarction, stent placement, or myocardial revascularization. Obesity was defined by a body mass index > 30 kg/m^2^ or if this diagnosis was reported in the medical record. Anemia was defined by an admission serum hemoglobin (Hb) value of < 13.0 g/dl in men or < 12.0 g/dl in women.

The other variables assessed were the degree of pulmonary involvement on chest computed tomography (mild, < 25%; moderate, between 25 and 50%; and severe, > 50%) and occurrence of additional organ dysfunction during hospitalization (pulmonary, circulatory, hepatic, and coagulation dysfunction). Pulmonary dysfunction was defined as a PaO2/FiO2 ratio < 400 or the need for mechanical ventilation, circulatory dysfunction was defined by the use of vasopressors, hepatic dysfunction was defined as serum levels of total bilirubin ≥ 1.2 mg/dl, and coagulation dysfunction was defined by platelet levels < 150,000/mm3 [18]. Finally, we assessed the use of corticosteroids, antibiotics and continuous infusion of heparin during hospitalization.

The KRT variables assessed were serum creatinine, urea, potassium, and bicarbonate up to 24 hours before the first KRT session; serum urea, potassium, and bicarbonate values (median) during the period when KRT was in use; and the method of KRT used: peritoneal dialysis (PD), intermittent hemodialysis (IHD), sustained low-efficiency dialysis (SLED), or continuous renal replacement therapy (CRRT). KRT was considered efficient if two or more of the following criteria were present after the commencement of KRT: mean urea values < 100 mg/dl, mean potassium values < 5.0 mEq/l, and mean bicarbonate values > 22 mEq/l. The outcomes assessed were hospital length of stay, death, and discharge with or without KRT dependence. The follow-up time was up to 90 days of hospitalization.

COVID-19 diagnosis was defined as a positive real-time polymerase chain reaction test or as a combination of respiratory symptoms and chest computed tomography with typical changes (peripheral and bilateral ground glass opacities, multifocal ground glass opacities of rounded morphology, and/or an inverted halo sign) [19].

### Statistical analysis

Categorical variables are described as frequencies. The Kolmogorov‒Smirnov test was used to define the distribution of quantitative variables. Quantitative variables with a parametric distribution are shown as the means and standard deviations, and those with a nonparametric distribution are presented as the medians and interquartile ranges. Frequencies were compared using the χ^2^ or Fisher’s exact test, as appropriate. Intergroup comparisons for quantitative variables were performed using Student’s t test and the Mann–Whitney U test for normally distributed and nonnormally distributed data, respectively.

Independent risk factors for lethality were identified by performing a logistic regression. The models were conducted using a stepwise and backward strategy. When there was a change in the estimate of a parameter greater than 10% with the exclusion of a variable, this variable remained in the model for adjustment. The goodness of fit was assessed using the Hosmer-Lemeshow test, and the significance of the variables was assessed using the Wald test.

For each wave, Model 1 included initial age (assessed at every five-year increment), sex (reference = woman) and number of comorbidities (1 or >1, reference = no comorbidities). Other variables included were (reference = absence) corticosteroid use, urea level >150 mg/dl, potassium level >5.0 mEq/l and bicarbonate level < 22 mEq/l at first KRT, efficient KRT and presence of at least two of the following types of organ dysfunction: hepatic, coagulation, pulmonary, and circulatory dysfunction. Model 2 was performed using the same variables but changing efficient KRT by the mean values assessed during the use of KRT of urea <100 mg/dl, potassium <5.0 mEq/l and bicarbonate > 22 mEq/l. Statistical analysis was performed using SPSS software, version 19.0 (SPSS Inc., Chicago, IL, USA). The significance level adopted was <0.05 and the confidence interval, 95%.

## Results

**Fig 1:**
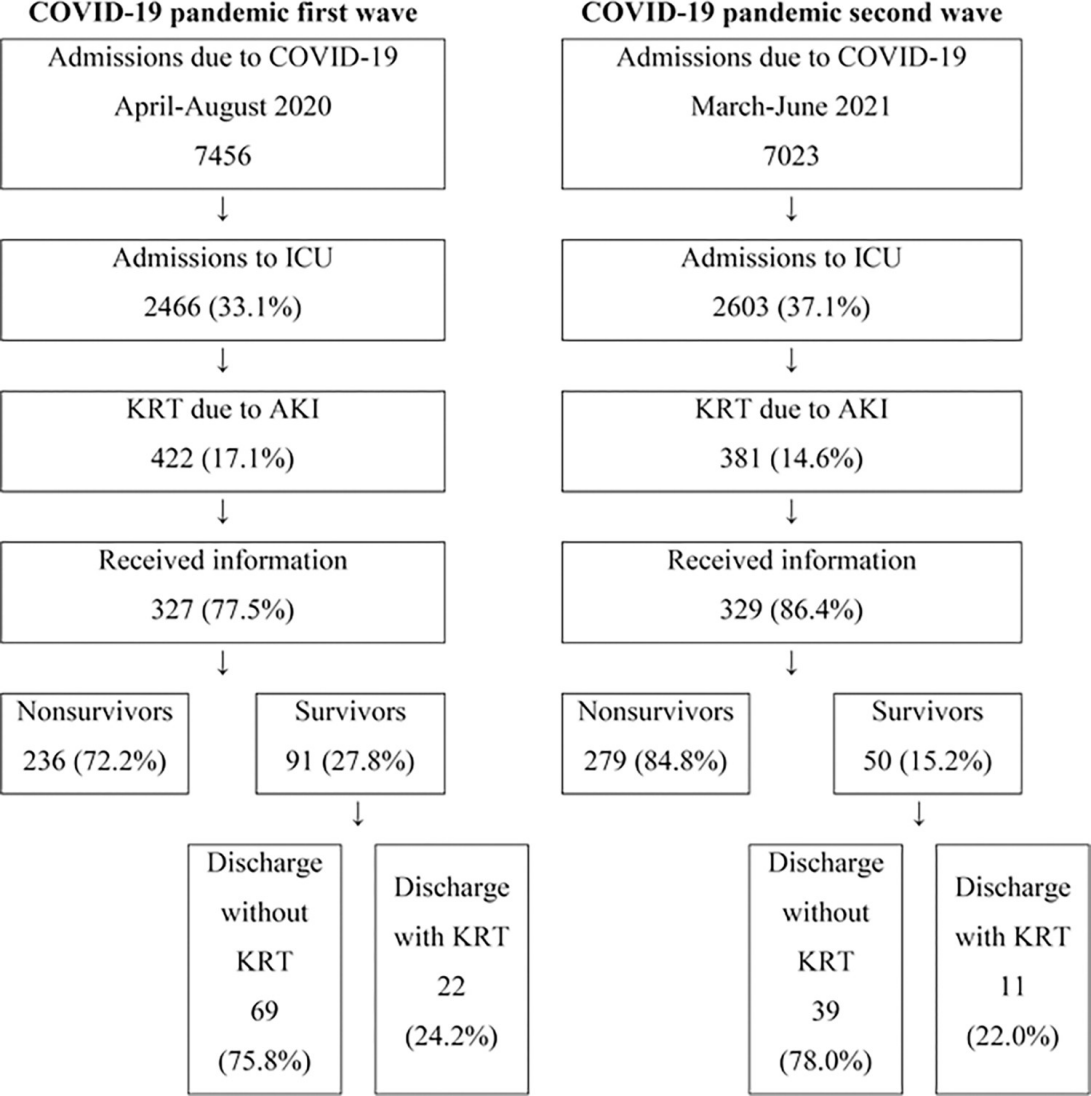
Flowchart of patient inclusion and outcomes. COVID-19, coronavirus disease 2019; ICU, intensive care unit; KRT, kidney replacement therapy; AKI, acute kidney injury.

During April-August 2020 and March-June 2021, 7,456 and 7,023 COVID-19 patients were admitted to the ten participating hospitals, respectively. There were 2,466 (33.1%) ICU admissions in the first pandemic wave and 2,603 (37.1%) in the second pandemic wave. KRT use due to AKI occurred among 422 (17.15%) patients in the first wave and among 381 (14.6%) in the second wave. This study received complete data from 327 (77.5%) and 329 (86.4%) patients in the first and second waves, respectively (Fig 1).

Second-wave patients were younger (61.4±13.7 vs. 63.8±13.6 years, p = 0.023) and had a lower frequency of diabetes (37.1% vs. 47.1%, p = 0.009) and obesity (29.5% vs. 40.0%, p = 0.007) than first-wave patients. Sex and ethnicity were similar in the two time periods. Second-wave patients sought health services later (duration from symptom onset: 7.1±5.0 vs. 5.6±3.9 days, p<0.001) and had a greater occurrence of coryza (19.5% vs. 9.8%, p<0.001), odynophagia (17.0% vs. 7.3%, p<0.001), expectoration (15.2% vs. 7.3%, p = 0.001) and ageusia (10.6% vs. 5.2%, p = 0.010) (Table 1).

**Table 1:** Patient characteristics.

Variable	First wave (n = 327)	Second wave (n = 329)	P-value
**Age, years**	**63.8 ±13.6**	**61.4 ±13.7**	**0.023**
Male, % (n)	68.5 (224)	67.2 (221)	0.716
Ethnicity			
White, % (n)	44.3 (145)	53.5 (176)	0.104
African descent, % (n)	21.1 (69)	15.8 (52)	
Asian, % (n)	2.4 (8)	1.8 (6)	
Unknown, % (n)	32.1 (105)	28.9 (95)	
Smoking, % (n)	18.0 (59)	12.8 (42)	0.061
Comorbidities			
Arterial hypertension, % (n)	67.6 (221)	61.4 (202)	0.098
** Diabetes mellitus, % (n)**	**47.1 (154)**	**37.1 (122)**	**0.009**
** Obesity, % (n)**	**40.0 (106)**	**29.5 (97)**	**0.007**
Chronic kidney disease, % (n)	19.9 (65)	16.8 (55)	0.304
Coronary artery disease, % (n)	16.5 (54)	12.8 (42)	0.174
Heart failure, % (n)	10.1 (33)	13.4 (44)	0.192
COPD, % (n)	8.3 (27)	9.1 (30)	0.695
Cancer, % (n)	4.0 (13)	4.0 (13)	0.987
Chronic liver disease, % (n)	2.4 (8)	0.6 (2)	0.055
**Number of comorbidities**			
** 0, % (n)**	**9.8 (32)**	**12.8 (42)**	**0.003**
** 1, % (n)**	**19.0 (62)**	**28.6 (94)**	
** 2 or more, % (n)**	**71.3 (233)**	**58.7 (193)**	
Public hospital, % (n)	39.4 (129)	41.3 (136)	0.622
Parameters at hospital admission			
**Duration of symptoms, days**	**5.6 ± 3.9**	**7.1 ± 5.0**	**<0.001**
Symptoms			
Dyspnea, % (n)	75.8 (248)	79.6 (262)	0.243
Cough, % (n)	74.3 (243)	68.4 (225)	0.093
Fever, % (n)	51.7 (169)	54.7 (180)	0.437
Diarrhea, % (n)	12.2 (40)	12.5 (41)	0.929
** Coryza, % (n)**	**9.8 (32)**	**19.5 (64)**	**<0.001**
** Odynophagia, % (n)**	**7.3 (24)**	**17.0 (56)**	**<0.001**
** Expectoration, % (n)**	**7.3 (24)**	**15.2 (50)**	**0.001**
Anosmia, % (n)	7.6 (25)	11.2 (37)	0.115
** Ageusia, % (n)**	**5.2 (17)**	**10.6 (35)**	**0.010**
Mean blood pressure (mmHg)	92 ± 19	90 ± 16	0.185
Oxygen saturation (%)	91 (87–95)	90 (86–95)	0.365

COPD, chronic obstructive pulmonary disease. Data are shown as means ± standard deviations, medians and interquartile ranges (p25-p75) or percentages

At hospital admission, second-wave patients showed lower total lymphocyte counts (807, IQR 508–1,299 vs. 955, IQR 697–1,460/mm^3^, p <0.001) and higher serum creatinine (1.25, IQR 0.93–2.20 vs. 1.11, IQR 0.90–1.75 mg/dl, p 0,011) and lower C-reactive protein values (13.2, IQR 6.1–22.6 vs. 15.9, IQR 7.6–25.7 mg/dl, p = 0.029). There was no difference between the two time periods regarding hemoglobin values, total leukocyte count, or pulmonary involvement on chest computed tomography. Second-wave patients had a greater use of mechanical ventilation (95.7% vs. 87.8%, p <0.001), had greater circulatory and pulmonary compromise (93.3% vs. 84.6%, p<0.001 and 85.4% vs. 78.3%, p = 0.018, respectively), and had a higher use of corticosteroids (86.0% vs. 61.3%, p<0.001) and a lower use of continuous heparin infusion (17.9% vs. 24.5%, p = 0.041). Second-wave patients started KRT with higher SCr (4.50, IQR 3.22–6.10 vs. 4.10, IQR 2.83–5.65 mg/dl, p = 0.011), urea (199, IQR 151–259 vs. 151, IQR 98–211 mg/dl, p<0.001) and serum potassium levels (5.3, IQR 4.5–5.9 vs. 4.8, IQR 4.2–5.5 mEq/l, p<0.001) than first-wave patients. The proportion of patients with efficient KRT was lower during the second wave (17.8% vs. 30.7%, p<0.001). The distribution of KRT modalities was similar in both groups. Lethality was higher during the second wave (84.8% vs. 72.2%, p<0.001), and KRT dependence upon hospital discharge was similar in both groups (Table 2).

**Table 2:** Laboratory results, used medications and outcomes.

Variable	First wave (n = 327)	Second wave (n = 329)	P-value
Laboratory values at hospital admission			
Hemoglobin (g/dl)	13.0 ± 2.1	13.1 ± 2.5	0.528
Total leukocyte count (n/mm^3^)	7965 (5512–11042)	8600 (5935–12145)	0.055
**Total lymphocytes (n/mm**^**3**^**)**	**955 (697–1460)**	**807 (508–1299)**	**<0.001**
Platelets x 10^3^ (n/mm^3^)	178.5 (141.0–227.0)	182.0 (137.5–233.5)	0.864
** Serum creatinine (mg/dl)**	**1.11 (0.90–1.75)**	**1.25 (0.93–2.20)**	**0.011**
D-dimer (ng/ml)	1.5 (0.7–9.7)	1.8 (0.7–22.8)	0.372
** C-reactive protein (mg/dl)**	**15.9 (7.6–25.7)**	**13.2 (6.1–22.6)**	**0.029**
Pulmonary involvement on CT			
Mild, % (n)	15.3 (43)	13.8 (45)	0.410
Moderate, % (n)	37.4 (105)	33.4 (109)	
Severe, % (n)	47.3 (133)	52.8 (172)	
**Mechanical ventilation, % (n)**	**87.8 (287)**	**95.7 (315)**	**<0.001**
Organ dysfunction			
** Hemodynamic, % (n)**	**84.6 (275)**	**93.3 (307)**	**<0.001**
** Pulmonary, % (n)**	**78.3 (256)**	**85.4 (281)**	**0.018**
Coagulation, % (n)	30.9 (101)	26.7 (88)	0.242
Hepatic, % (n)	16.1 (50)	16.4 (54)	0.922
**Number of types of organ dysfunction**			
** 0, % (n)**	**8.6 (28)**	**1.8 (6)**	**0.001**
** 1, % (n)**	**13.5 (44)**	**14.3 (47)**	
** 2 or more, % (n)**	**78.0 (255)**	**83.9 (276)**	
Medications			
** Vasopressors, % (n)**	**84.6 (275)**	**93.3 (307)**	**<0.001**
Antimicrobials, % (n)	97.9 (282)	97.9 (322)	0.969
** Corticosteroids, % (n)**	**61.3 (200)**	**86.0 (282)**	**<0.001**
** Continuous heparin infusion, % (n)**	**24.5 (79)**	**17.9 (59)**	**0.041**
Laboratory values on KRT indication day			
** Creatinine (mg/dl)**	**4.10 (2.83–5.65)**	**4.50 (3.22–6.10)**	**0.011**
** Urea (mg/dl)**	**151 (98–211)**	**199 (151–259)**	**<0.001**
** Potassium (mEq/l)**	**4.8 (4.2–5.5)**	**5.3 (4.5–5.9)**	**<0.001**
Bicarbonate (mEq/l)	22.7 ± 5.7	22.5 ± 5.6	0.705
Mean laboratory values during KRT			
Creatinine (mg/dl)	3.63 (2.10–5.10)	3.50 (2.30–5.05)	0.949
** Urea (mg/dl)**	**123 (86–178)**	**152 (110–211)**	**<0.001**
Potassium (mEq/l)	4.7 (4.2–5.4)	4.8 (4.2–5.5)	0.251
Bicarbonate (mEq/l)	22.8 ± 5.1	23.1 ± 6.0	0.410
**KRT efficiency, % (n)**	**30.7 (92)**	**17.8 (55)**	**<0.001**
KRT method			
IHD, % (n)	54.1 (177)	55.9 (184)	0.182
SLED, % (n)	8.0 (26)	0.9 (3)	
CRRT, % (n)	19.9 (65)	15.2 (50)	
PD, % (n)	0,0 (0)	0,0 (0)	
Combination of methods, % (n)	18.0 (59)	28.0 (92)	
Time on KRT (days)	6 (3–15)	8 (4–16)	0.078
Outcomes			
Length of hospitalization (days)	24.1 ± 18.0	26.7 ± 23.2	0.104
** Death, % (n)**	**72.2 (236)**	**84.8 (279)**	**<0.001**
Discharge with KRT, % (n)	24.2 (22)	22.0 (11)	0.770

CT, computed tomography. KRT, kidney replacement therapy. IHD, intermittent hemodialysis. SLED, sustained low efficiency dialysis. CRRT, continuous renal replacement therapy. PD, peritoneal dialysis. Data are shown as the means ± standard deviations, medians and interquartile ranges (p25-p75) or percentages.

### Risk factors for death: Univariate analysis

Survivors from the first wave had a lower frequency of two or more types of organ dysfunction (64.8% vs. 83.1%, p<0.001), lower use of corticosteroids (44.0% vs. 68.1%, p<0.001) and more patients with efficient KRT (40.7% vs. 26.9%, p = 0.021) than nonsurvivors (Table 3). Survivors from the second wave were younger (55.8±12.5 vs. 62.4±13.7 years, p = 0.002), had a lower frequency of two or more types of organ dysfunction (78.0% vs. 84.9%, p<0.001) and lower median urea (128, IQR 102–160 mg/dl vs. 163, IQR 117–217 mg/dl, p = 0.001) and potassium (4.3, IQR 4.0–4.9 mEq/l vs. 4.9, IQR 4.3–5.6 mEq/l, p<0.001) values during KRT when compared to nonsurvivors (Table 3).

**Table 3:** Comparison between survivors and nonsurvivors.

	First wave (n = 327)			Second wave (n = 329)		
Variable	Survivors (n = 91)	Nonsurvivors (n = 236)	P-value	Survivors (n = 50)	Nonsurvivors (n = 279)	P-value
**Age, years**	69.9 ± 13.4	64.2 ± 13.7	0.450	**55.8 ± 12.5**	**62.4 ± 13.7**	**0.002**
Number of comorbidities						
0, % (n)	12.1 (11)	13.6 (32)	0.908	18.0 (9)	11.5 (32)	0.321
1, % (n)	22.0 (20)	20.3 (48)		22.0 (11)	29.4 (82)	
2 or more, % (n)	65.9 (60)	66.1 (156)		60.0 (30)	59.1 (165)	
Pulmonary injury						
Mild, % (n)	11.8 (10)	16.8 (33)	0.210	16.0 (8)	13.4 (37)	0.306
Moderate, % (n)	44.7 (38)	34.2 (67)		24.0 (12)	35.1 (97)	
Severe, % (n)	43.5 (37)	49.0 (96)		60.0 (30)	51.4 (142)	
**Number of types of organ dysfunction**						
** 0, % (n)**	**18.7 (17)**	**4.7 (11)**	**<0.001**	**10.0 (5)**	**0.4 (1)**	**0.001**
** 1, % (n)**	**16.5 (15)**	**12.3 (29)**		**12.0 (6)**	**14.7 (41)**	
** 2 or more, % (n)**	**64.8 (59)**	**83.1 (236)**		**78.0 (39)**	**84.9 (237)**	
Medications						
** Vasopressors, % (n)**	**11.4 (9)**	**52.7 (108)**	**<0.001**	**20.0 (10)**	**63.1 (176)**	**<0.001**
** Corticosteroids, % (n)**	**44.0 (40)**	**68.1 (160)**	**<0.001**	88.0 (44)	85.6 (238)	0.654
Continuous heparin infusion, % (n)	22.2 (20)	25.3 (59)	0.561	24.0 (12)	16.8 (47)	0.225
Mean laboratory values during KRT						
Creatinine (mg/dl)	3.21 (1.80–4.90)	3.74 (2.30–5.20)	0.061	2.92 (2.20–4.00)	3.55 (2.39–5.20)	0.080
Urea (mg/dl)	108.0 (77.0–164.0)	129 (90–184)	0.056	**128 (102–160)**	**163 (117–217)**	**0.001**
** Potassium (mEq/l)**	**4.5 (4.0–5.0)**	**4.8 (4.2–5.5)**	**<0.001**	**4.3 (4.0–4.9)**	**4.9 (4.3–5.6)**	**<0.001**
** Bicarbonate (mEq/l)**	**24.0 ± 5.6**	**22.3 ± 4.9**	**0.012**	24.1 ± 6.0	23.0 ± 6.0	0.209
**Efficient KRT, % (n)**	**40.7 (33)**	**26.9 (59)**	**0.021**	20.8 (10)	17.2 (45)	0.550
**Time on KRT (days)**	**15 (7–24)**	**4 (2–11)**	**<0.001**	**22 (10–32)**	**7 (3–13)**	**<0.001**
**Hospitalization length (days)**	**37 ± 19**	**19 ± 15**	**<0.001**	**55 ± 38**	**22 ± 15**	**<0.001**

COPD, chronic obstructive pulmonary disease; KRT, kidney replacement therapy. Data are shown as means ± standard deviations, medians and interquartile ranges (p25-p75) or percentages

### Risk factors for death: Multivariate analysis

The independent variables associated with death in the final models of multiple logistic regression are shown in Table 4.

**Table 4:** Risk factors associated with lethality.

	Adjusted OR ^a^ (95% CI)	P-value
First wave		
Model 1^a^		
Use of corticosteroids	2.96 (1.58–5.57)	<0.001
Presence of 2 or more types of organ dysfunction ^b^	3.56 (1.75–7.25)	<0.001
Efficient KRT ^c^	0.28 (0.15–0.54)	<0.001
Model 2^a^		
Use of corticosteroids	2.85 (1.54–5.29)	<0.001
Presence of 2 or more types of organ dysfunction ^b^	3.20 (1.61–6.34)	<0.001
Mean potassium level during KRT <5.0 mEq/l	0.35 (0.18–0.69)	0.003
Second wave		
Model 1^a^		
Age (5-year increments)	1.29 (1.13–1.46)	<0.001
Potassium level on first KRT day >5.0 mEq/l	3.53 (1.75–7.10)	<0.001
Efficient KRT ^c^	0.48 (0.25–0.94)	0.033
Model 2^a^		
Age (5-year increments)	1.28 (1.13–1.45)	<0.001
Potassium level on first KRT day >5.0 mEq/l	3.11 (1.52–6.34)	0.002
Mean potassium level during KRT <5.0 mEq/l	0.35 (0.16–0.76)	0.008

OR, Odds ratio; CI, confidence interval; KRT, kidney replacement therapy.

^a^ Significant variable in the final multiple regression model. All variables were adjusted for those included in the initial model: age, sex, number of comorbidities (none, one or >one), use of corticosteroids, presence of 2 or more types of organ dysfunction and potassium level on first KRT day >5.0 mEq/l. In Model 1, the variable efficient KRT was added, and in Model 2, this variable was changed to mean values of urea <100 mg/dl, potassium <5.0 mEq/l and bicarbonate >22 mEq/l during KRT.

^b^ Presence of at least two of the following types of organ dysfunction: hepatic, coagulation, pulmonary and circulatory dysfunction.

^c^ Presence of at least two of the following mean serum values evaluated during KRT: urea <100 mg/dl, potassium <5.0 mEq/l and bicarbonate > 22 mEq/l

#### First wave

In Model 1, which included efficient KRT, death was positively associated with corticosteroid use and the presence of two or more types of organ dysfunction and negatively associated with efficient KRT. In Model 2, efficient KRT was replaced by mean values of urea < 100 mg/dl, potassium <5.0 mEq/l and bicarbonate >22 mEq/L during KRT. In this model, death was positively associated with corticosteroid use and the presence of two or more types of organ dysfunction and negatively associated with mean values of potassium <5.0 mEq/l during KRT.

#### Second wave

In Model 1, death was positively associated with older age and potassium levels >5.0 mEq/l in the first KRT session and negatively associated with efficient KRT. In Model 2, death was also positively associated with older age and potassium levels >5.0 mEq/l in the first KRT session and negatively associated with mean values of potassium <5.0 mEq/l during KRT.

### Risk factors for KRT dependency at discharge: Univariate analysis

Patients discharged without KRT dependency in the first wave showed lower admission SCr values (1.06, IQR 0.87–1.52 vs. 3.00, IQR 1.25–7.25 mg/dl, p<0,001) and lower urea values on the day of first KRT indication (130, IQR 82–193 vs. 179, IQR 118–244 mg/dl, p = 0.029) than KRT-dependent patients on discharge.

Patients discharged without KRT dependency in the second wave were younger (53.9±11.3 vs. 62.3±14.6 years, p = 0.038), had higher hospital admission hemoglobin (13.7±2.2 vs. 10.7±2.7 g/dl, p<0.001) and lower hospital admission SCr (1.30, IQR 1.00–2.69 vs. 3.30, IQR 1.28–7.13 mg/dl, p = 0.030) values, a higher number of types of organ dysfunction (p = 0.021) and higher serum potassium and bicarbonate levels on the day when KRT was indicated (potassium: 4.9, IQR 4.5–5.9 vs. 4.5, IQR 4.0–4.8 mEq/l, p = 0.037; bicarbonate: 23.0±5.0 vs. 18.1±5.7 mEq/l, p = 0.010, respectively). Last, they had higher serum bicarbonate levels during KRT (25.2±5.5 vs. 20.1±6.2 mEq/l, p = 0.014) (Table 5).

**Table 5:** Comparison between patients discharged with or without kidney replacement therapy.

	First wave (n = 91)			Second wave (n = 50)		
Variable	Discharge without KRT (n = 69)	Discharge with KRT (n = 22)	P-value	Discharge without KRT (n = 39)	Discharge with KRT (n = 11)	P-value
**Age, years**	62.3 ± 13.9	63.8 ± 11.7	0.734	**53.9 ± 11.3**	**62.3 ± 14.6**	**0.038**
Number of comorbidities						
0, % (n)	13.0 (9)	9.1 (2)	0.140	23.1 (9)	0.0 (0)	0.061
1, % (n)	26.1 (18)	9.1 (2)		23.1 (9)	18.2 (2)	
2 or more, % (n)	60.9 (42)	81.8 (18)		53.8 (21)	81.8 (9)	
Laboratory values at admission						
Hemoglobin (g/dl)	13.0 ± 2.1	13.5 ± 1.9	0.414	**13.7 ± 2.2**	**10.7 ± 2.7**	**<0.001**
Total leukocytes (n/mm^3^)	9220 (6290–13185)	7400 (5210–9310)	0.062	7950 (5,070–11,848)	10800 (6190–19100)	0.125
Platelets x 10^3^ (n/mm^3^)	192.5 (150.5–266.2)	161.0 (128.0–260.0)	0.216	172.5 (143.0–212.0)	172.5 (126.5–229.5)	0.990
** Serum creatinine (mg/dl)**	**1.06 (0.87–1.52)**	**3.00 (1.25–7.25)**	**<0.001**	**1.30 (1.00–2.69)**	**3.30 (1.28–7.13)**	**0.030**
**Number of types of organ dysfunction**						
** 0, % (n)**	76.8 (53)	54.5 (12)	0.503	5.1 (2)	27.3 (3)	**0.021**
** 1, % (n)**	72.5 (50)	54.5 (12)		7.7 (3)	27.3 (3)	
** 2 or more, % (n)**	20.3 (14)	31.8 (7)		87.2 (34)	45.5 (5)	
Laboratory values at first KRT indication						
Creatinine (mg/dl)	4.00 (2.77–6.15)	4.60 (3.29–7.05)	0.342	5.0 (3.70–6.30)	5.35 (3.56–7.32)	0.990
** Urea (mg/dl)**	**130 (82–193)**	**179 (118–244)**	**0.029**	184 (119–240)	165 (129–207)	0.440
** Potassium (mEq/l)**	4.5 (3.9–5.2)	4.9 (4.2–5.4)	0.075	**4.9 (4.5–5.9)**	**4.5 (4.0–4.8)**	**0.037**
** Bicarbonate (mEq/l)**	24.0 ± 6.3	21.0 ± 5.9	0.056	**23.0 ± 5.0**	**18.1 ± 5.7**	**0.010**
Mean laboratory values during KRT						
Creatinine (mg/dl)	2.89 (1.80–4.67)	3.80 (2.43–2.25)	0.135	2.80 (2.0–3.90)	3.75 (2.83–5.91)	0.054
Urea (mg/dl)	111 (77–162)	98 (75–166)	0.816	124 (102–163)	130 (85–151)	0.836
Potassium (mEq/l)	4.4 (4.0–4.9)	4.8 (3.9–5.6)	0.211	4.3 (4.0–4.9)	4.4 (3.6–4.9)	0.566
** Bicarbonate (mEq/l)**	24.5 ± 5.9	22.6 ± 4.5	0.180	**25.2 ± 5.5**	**20.1 ± 6.2**	**0.014**
Efficient KRT, % (n)	38.3 (23)	47.6 (10)	0.456	18.4 (7)	30.0 (3)	0.437
Time on KRT (days)	15 (7–23)	15 (7–36)	0.455	21 (9–31)	28 (11–43)	0.189
Hospitalization length (days)	38 ± 17	31 ± 23	0.109	58 ± 39	45 ± 35	0.312

KRT, kidney replacement therapy. Data are shown as the means ± standard deviations, medians and interquartile ranges (p25-p75) or percentages.

## Discussion

This multicenter study performed among critically ill patients hospitalized in the metropolitan area of Sao Paulo, Brazil, showed that patients who developed COVID-19-associated AKI and received KRT in the second wave of the pandemic were younger and had fewer comorbidities than first-wave patients but had a greater number of types of organ dysfunction. The duration from symptom onset to hospitalization was longer among second-wave patients. These patients received less efficient KRT and had higher lethality rates.

To our knowledge, no previous study has been specifically designed to investigate the present research question. The little available information has dissimilarities with our findings. A study from Ontario, Canada, showed that second-wave patients who developed COVID-19-associated AKI and required KRT, compared with first-wave ones, were older, had more comorbidities and initiated KRT later after the beginning of COVID-19 symptomatology [20]. A single-center study in England found a lower risk of AKI and need for KRT in the second wave, although these patients were older and had higher frailty scores [21].

We observed that younger individuals with fewer comorbidities were mostly affected in the second wave of the pandemic, a finding consistent with the results of a large COVID-19 Brazilian cross-sectional study comparing the two pandemic waves [22]. A possible explanation for this finding is the vaccination strategy adopted in Brazil, which prioritized older people and individuals with comorbidities. It is well known that the main effect of vaccines against COVID-19 is the prevention of progression to severe forms of the disease, reducing hospitalizations and deaths [23]. During the inclusion period of second-wave patients in our study, Brazilian vaccination coverage slightly increased from 0.9% to 12.4%.2 As a means of comparison, during the same period, vaccination coverage was 55% in Chile and 49% in the USA and United Kingdom [2].

The higher frequency of mechanical ventilation and vasopressor use observed among our second-wave patients contrasts with those in studies conducted in high-income countries [24–27]. This discrepancy might be attributed to variations in the ability of health care systems to respond to the pandemic and to the effectiveness of preventive measures, including population testing patterns, social distancing practices, and vaccination rates. As of June 30, 2021, the United Kingdom, USA, and Chile had accumulated test rates of 3,012, 1,401, and 858 per 1,000 inhabitants, respectively, while Brazil had a significantly lower rate of only 249 [2]. Early disease diagnosis can have several benefits, including facilitating prompt isolation of affected individuals, reducing the time taken to seek medical assistance, and minimizing the dissemination of highly pathogenic viral strains. In fact, the more prolonged duration between symptom onset and hospitalization observed among our second-wave patients could potentially be attributed to challenges in accessing the health care system. In addition, a plausible explanation for the increased severity observed among patients during the second wave in Sao Paulo is the predominance of the Gamma (or P1) virus variant in Brazil during the study inclusion period [28]. Some studies have shown that this variant is associated with higher transmissibility, immune response evasion and resistance to neutralization by serum from vaccinated individuals and subsequently higher severity and hospitalization risk [29–32].

A key observation of our study was the association between KRT efficiency and lethality. Our results also suggested that delaying the initiation of KRT, as measured by serum potassium immediately before KRT, might be associated with higher lethality. Less than one-third and one-fifth of the patients received efficient KRT in the first and second waves, respectively. Differences between prescribed and offered KRT doses have been previously described, and it is well known that subdoses of KRT are associated with higher lethality [33–35]. Indeed, our study showed that the achievement of adequate markers of efficient KRT was associated with reduced lethality rates in both pandemic waves, especially with regard to serum potassium levels [36]. The decreased efficiency of KRT observed during the second wave might be attributed to several factors. These include greater disease severity along with hemodynamic instability, as well as limited availability of intra-hospitalar resources. This scarcity encompasses a reduced availability of skilled health care professionals capable of ensuring appropriate KRT and an inability to provide continuous forms of KRT to patients with hemodynamic instability [37].

The association between corticosteroid use and the lethality rate during the first wave might be related to a greater severity of the disease not detected in multiple regression or by inaccurate treatment resulting from the lack of knowledge about the disease during a period when the evidence on the effectiveness of corticosteroids was still under investigation. Unfortunately, it was not possible to obtain the medical indication for the use of corticosteroids and the day of it initiation.

The higher lethality of our COVID-19 patients with AKI treated with KRT during the second wave is consistent with that in epidemiological studies conducted in low- and low-middle-income countries (African countries, Mexico, India) [38–40] but not in high-income countries (USA, United Kingdom, and Spain) [41–43]. Such results suggest that lower-income countries exhibit less resilience in coping with the long-term effects of the pandemic.

The limitations of this study are its retrospective design, the lack of information on kidney function recovery after hospital discharge and the unavailability of following data: the number of KRT sessions, the total hours of CRRT, the dialysis anticoagulation prescriptions, and the indications for KRT onset. In addition, it was not possible to obtain other measures of dialysis quality, such as Kt/V, due to the retrospective design of the study and because this assessment is not routine in the participating hospitals. Indeed, key performance indicators for assessing efficacy of KRT in AKI have not been consistently determined and universally tested [44]. Therefore, we chose the parameters considering that they fulfill the criteria for assessing the efficiency of KRT in the context of the studied patients. Lastly, we excluded participants from three hospitals from the study because these institutions had not sent information on the second wave of the pandemic. Despite this, compared to the patients included in the study, these individuals had no significant differences in age, in the prevalence of the main comorbidities, in the incidence of organic dysfunctions and in their outcomes (S1 File).

## Conclusions

In summary, the lethality of patients with COVID-19-associated AKI treated with KRT in the São Paulo megalopolis was higher in the second wave than in the first wave. Health care service accessibility, the availability of adequate intra-hospitalar resources (including offered KRT efficiency and quality), the pace of vaccination coverage and targeted strategies for specific groups, and the presence of specific variants of the causal agent should be considered possible causes for the observed results. It is important to highlight that the majority of these factors are potentially modifiable, implying that interventions to address these gaps in health care system care have a greater likelihood of mitigating the adverse outcomes observed in this study.

## Supporting information

S1 File (DOCX)
