# Supplementary material for: Critically ill patients with COVID-19-associated acute kidney injury treated with kidney replacement therapy: Comparison between the first and second pandemic waves in São Paulo, Brazil
Source: PLoS One. 2023 Nov 3;18(11):e0293846. doi: 10.1371/journal.pone.0293846 (PMC10624321; doi:10.1371/journal.pone.0293846)
Supplement: S1 File — (DOCX) [file pone.0293846.s001.docx]

**Critically ill patients with COVID-19-associated acute kidney injury treated with kidney replacement therapy: Comparison between the first and second pandemic waves in São Paulo, Brazil**

**Supporting information**

Table 6S. Characteristics of first wave patients included and excluded in the study.

| Variable | Patients included  (n=327) | Patients excluded  (n=48) | P-value |
| --- | --- | --- | --- |
| Age, years | 63.8 ±13.6 | 64.8 (54.7-71.5) | 0.934 |
| Male, % (n) | 68.5 (224) | 70.8 (34) | 0.745 |
| Comorbidities |  |  |  |
| Arterial hypertension, % (n) | 67.6 (221) | 70.8 (34) | 0.652 |
| Diabetes mellitus, % (n) | 47.1 (154) | 33.3 (16) | 0.074 |
| Obesity, % (n) | 40.0 (106) | 37.0 (10) | 0.764 |
| Chronic kidney disease, % (n) | 19.9 (65) | 10.4 (5) | 0.116 |
| Coronary artery disease, % (n) | 16.5 (54) | 10.4 (5) | 0.279 |
| Public hospital, % (n) | 39.4 (129) | 39.6 (19) | 0.986 |
| Parameters at hospital admission |  |  |  |
| Duration of symptoms, days | 5.6 ± 3.9 | 8.0 ± 5.1 | <0.001 |
| Mean blood pressure (mmHg) | 92 ± 19 | 90 ± 15 | 0.632 |
| Oxygen saturation (%) | 91 (87-95) | 87 (91-95) | 0.042 |

Data are shown as means ± standard deviations, medians and interquartile ranges (p25-p75) or percentages.

Table 7S. Laboratory results, used medications and outcomes of first wave patients included and excluded in the study.

| Variable | Patients included  (n=327) | Patients excluded  (n=48) | P-value |
| --- | --- | --- | --- |
| Laboratory values at hospital admission |  |  |  |
| Hemoglobin (g/dl) | 13.0 ± 2.1 | 12.8 ± 2.2 | 0.518 |
| Total leukocyte count (n/mm^3^) | 7965 (5512-11042) | 9100 (5500-12110) | 0.320 |
| Total lymphocytes (n/mm^3^) | 955 (697-1460) | 979 (720-1428) | 0.978 |
| Platelets x 10³ (n/mm^3^) | 178.5 (141.0-227.0) | 184.5 (139.3-268.8) | 0.517 |
| Serum creatinine (mg/dl) | 1.11 (0.90-1.75) | 1.20 (0.90-1.90) | 0.487 |
| Mechanical ventilation, % (n) | 87.8 (287) | 93.8 (45) | 0.224 |
| Organ dysfunction |  |  |  |
| Hemodynamic, % (n) | 84.1 (275) | 87.5 (42) | 0.543 |
| Pulmonary, % (n) | 78.3 (256) | 81.3 (39) | 0.640 |
| Coagulation, % (n) | 30.9 (101) | 27.1 (13) | 0.593 |
| Hepatic, % (n) | 16.1 (50) | 13.3 (6) | 0.631 |
| Medications |  |  |  |
| Vasopressors, % (n) | 84.6 (275) | 87.5 (42) | 0.601 |
| Antimicrobials, % (n) | 97.9 (282) | 90.9 (40) | 0.032 |
| Corticosteroids, % (n) | 61.3 (200) | 54.2 (26) | 0.342 |
| Continuous heparin infusion, % (n) | 24.5 (79) | 21.7 (10) | 0.687 |
| Laboratory values on KRT indication day |  |  |  |
| Creatinine (mg/dl) | 4.10 (2.83-5.65) | 3.90 (3.33-5.20) | 0.869 |
| Urea (mg/dl) | 151 (98-211) | 169 (109-211) | 0.475 |
| Potassium (mEq/l) | 4.8 (4.2-5.5) | 4.7 (4.1-5.4) | 0.595 |
| Bicarbonate (mEq/l) | 22.7 ± 5.7 | 21.6 ± 5.1 | 0.249 |
| Mean laboratory values during KRT |  |  |  |
| Creatinine (mg/dl) | 3.63 (2.10-5.10) | 3.62 (2.34-5.45) | 0.999 |
| Urea (mg/dl) | 123 (86-178) | 134 (100-161) | 0.678 |
| Potassium (mEq/l) | 4.7 (4.2-5.4) | 4.7 (3.8-5.1) | 0.212 |
| Bicarbonate (mEq/l) | 22.8 ± 5.1 | 21.8 ± 4.5 | 0.263 |
| KRT efficiency, % (n) | 30.7 (92) | 21.4 | 0.219 |
| Outcomes |  |  |  |
| Death, % (n) | 72.2 (236) | 75.0 (36) | 0.682 |
| Discharge with KRT, % (n) | 24.2 (22) | 8.3 (1) | 0.174 |

KRT, kidney replacement therapy. Data are shown as the means ± standard deviations, medians and interquartile ranges (p25-p75) or percentages.
